# Early Localization of Bronchogenic Cancerous/Precancerous Lesions With Lung Imaging Fluorescence Endoscope

**DOI:** 10.1155/DTE.3.197

**Published:** 1997

**Authors:** N. Ikeda, K. Kim, T. Okunaka, K. Furukawa, T. Furuya, M. Saito, C. Konaka, H. Kato, Y. Ebihara

**Affiliations:** 1 Department of Surgery Tokyo Medical College Hospital 6-7-1, Nishishinjuku Shinjuku-ku Tokyo 160 Japan; 2 Department of Pathology II Tokyo Medical College Hospital 6-7-1, Nishishinjuku Shinjuku-ku Tokyo 160 Japan

## Abstract

The result of the clinical trial using the lung imaging fluorescence endoscope (LIFE) was reported. A total of 77 biopsy confirmed sites from 30 patients were evaluated. The sensitivity for metaplasia detection by the LIFE system was 96% compared to 28% by conventional bronchoscopy. The LIFE system was found to be useful in detecting subtle cancerous/precancerous lesions.


*Diagnostic and Therapeutic Endoscopy*, 1997, Vol. 3, pp. 197–201
Reprints available directly from the publisher
Photocopying permitted by license only

© 1997 OPA (Overseas Publishers Association)
Amsterdam B.V. Published in The Netherlands
by Harwood Academic Publishers
Printed in Singapore

### Early Localization of Bronchogenic Cancerous/ Precancerous Lesions with Lung Imaging Fluorescence Endoscope

N. IKEDA[1*], K. KIM[1], T. OKUNAKA[1], K. FURUKAWA[1], T. FURUYA[1], M. SAITO[1], C. KONAKA[1], H. KATO[1] and Y. EBIHARA[2]

[1]*Department of Surgery, Department of Pathology II*[2]*, Tokyo Medical College Hospital, 6-7-1, Nishishinjuku, Shinjuku-ku, Tokyo 160, Japan*

*(Received 13 July 1996; in final form 27 September 1996)*

**The result of the clinical trial using the lung imaging fluorescence endoscope (LIFE) was reported. A total of 77 biopsy confirmed sites from 30 patients were evaluated. The sensitivity for metaplasia detection by the LIFE system was 96% compared to 28% by conventional bronchoscopy. The LIFE system was found to be useful in detecting subtle cancerous/precancerous lesions.**

*Keywords:* Fluorescence endoscope, squamous metaplasia, early lung cancer, early detection

### INTRODUCTION

The increasing numbers of lung cancer deaths throughout the world are mainly due to the detection of the disease at late stage. Since lung cancer is highly malignant, its prevention and early detection are essential to decrease the death rate. However, the detection of early lung cancer, especially carcinoma *in situ*, is a diagnostic challenge for bronchoscopists because these lesions do not always show endoscopically visible changes [1]. Photodynamic diagnosis is a modality to enhance the lesions using photosensitizer which has an affinity to tumor tissue [2–4]. However, photosensitizers can have side effects and are not commonly used. To overcome this problem, fluorescence detection system has been created. The lung imaging fluorescence endoscope (LIFE) system was developed to detect precancerous and CIS lesions, based on differences in normal and abnormal tissue auto-fluorescence [5-7]. In this study, the authors will report the result of a clinical trial using the LIFE system performed at our institution.

*Corresponding Author: Norihiko Ikeda, Department of Surgery, Tokyo Medical College Hospital, 6-7-1, Nishishinjuku, Shinjuku-ku, Tokyo 160, Japan

Table I Histological classification of the biopsied sites (77 sites / 30 cases)

| | |
|---|---|
| Invasive cancer | 13 sites |
| Carcinoma *in situ* | 3 sites |
| Squamous metaplasia | 25 sites |
| Normal/Inflammation | 36 sites |

Table II Endoscopy and biopsy results.

| | | LIFE | |
|---|---|---|---|
| | | negative | positive |
| BF | negative | 0 | 0 |
| | positive | 0 | 3 |

Carcinoma *in situ* (3 sites)

### MATERIAL AND METHOD

### Lung Imaging Fluorescence Endoscope (LIFE)[5]

Normal bronchial tissue fluoresces green when excited by blue light, but abnormal tissue lacks green auto-fluorescence [8,9]. Sophisticated application of this principle produced the LIFE system [8]. The LIFE system consists of a light source (helium-cadmium laser, 442 nm) and image intensifier (CCD camera with green and red filters). Two images at different (red and green) wavelengths are simultaneously captured. The images are combined and processed by an imaging board using a special algorithm that separates images of normal tissue from those from cancerous/precancerous tissue. The autofluorescence image is displayed in real time on image monitor.

### Subjects

A total of 30 subjects were studied and 77 sites were biopsied. Table I shows the histological result of the biopsies: invasive cancer 13, CIS 3, atypical squamous metaplasia 25, normal and chronic inflammation 36, respectively. These cases were referred to Tokyo Medical College Hospital because of a strong suspicion of lung cancer or abnormal sputum cytology findings. The number of examinations using the LIFE system were restricted to thirty per institution in this clinical trial by the Japanese Ministry of Health and Welfare.

### Clinical Protocol

Conventional fiberoptic bronchoscopic examination (BF 20 or BF 1T-30, Olympus Optical Co., Tokyo, Japan) was first performed and areas with abnormal findings (suspicious for cancer or metaplasia) were noted for subsequent biopsy. The fluorescence examination was performed. Any abnormal areas recognized by conventional bronchoscopy or by the LIFE system were biopsied. Procedures were carried out under local anesthesia and no additional sedation was needed in any case nor was there any complaint of side effects. All biopsy specimens were interpreted in our Department of Pathology, and pathological diagnosis was the gold standard for the comparison of diagnostic accuracy of conventional bronchoscopy and LIFE.

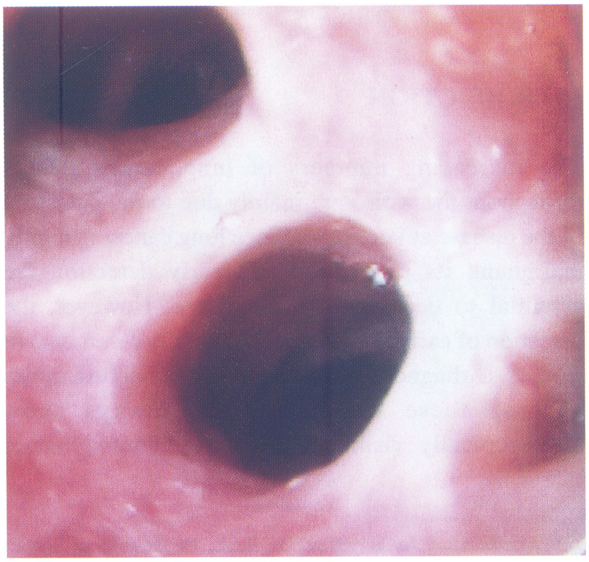

FIGURE 1A Right B6 under conventional bronchoscopy. Thickening of bifurcation was found.

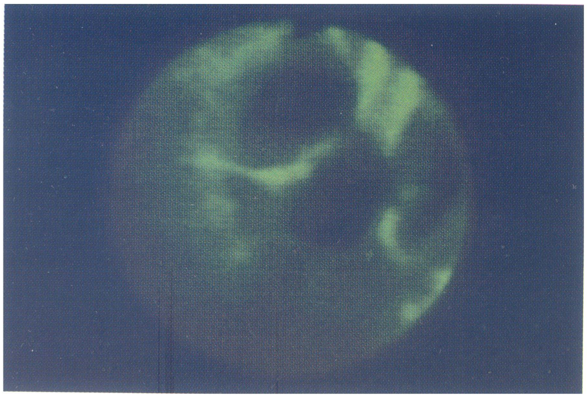

FIGURE 1B Same area as Figure 1a under LIFE examination. Carcinoma *in situ* appeared cold image.

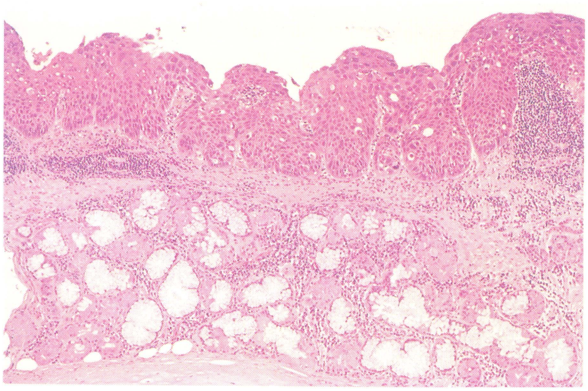

FIGURE 1C Biopsy revealed squamous cell carcinoma.

### RESULTS

Table II shows the biopsy and endoscopy (conventional/LIFE) results. Of the 77 sites biopsied in 30 subjects, cancer was detected at 16 sites (invasive cancer 13, CIS 3). The LIFE diagnosed all 16 of them and had no false negatives. The white light bronchoscopy diagnosed 15 of the 16 cancers and had 1 false negative (6%). This was a recurrent lesion in the surgical stump after right upper lobectomy which seemed to be merely a normal post-operative change by conventional bronchoscopy. A total of 3 CIS lesions were discovered by both methods but the extent and margin of the tumor were more clearly observed under fluorescence examination. Figure 1a shows the brochofiberscopic finding of a CIS lesion in right B6. Thickening of the bifurcation can be seen. Figure 1b shows the LIFE imaging of the same lesion. Biopsy of this area showed carcinoma *in situ* (Fig. 1c). Normal fluorescence decreased at the site of CIS. Metaplasia (moderate or more severe in all cases) was detected at 25 sites. The sensitivity for metaplasia detection by conventional bronchoscopy was 28% (7/25) compared to 96% (24/25) by the LIFE system (Table II). Figure 2a shows a right basal bronchus which seems to be normal on conventional bronchoscopy. Figure 2b shows the LIFE image of the same site. A cold spot can be detected in the bifurcation of B8 and biopsy revealed metaplasia with moderate atypia (Fig. 2c). In relation to specificity, the LIFE system had a slightly higher false positive (16/36, 44%) rate than the white light bronchoscopy (14/36, 39%) but the difference was not statistically significant. The definition of positive was atypical metaplasia or cancer.

### DISCUSSION

Sputum cutology examination is often the first method to detect central type early cancer or metaplasia, but we sometimes fail to localize the lesion. Such lesions are so thin that they do not show endoscopically-visible mucosal changes [1,4]. The concept of fluorescence endoscopy depends on the fact that an abnormal area has different autofluorescence from that of normal areas. The normal area of the bronchus shows green autofluorescence when excited by blue light, but abnormal areas such as cancer or metaplasia show cold spots due to decreased green auto-fluorescence [6]. Several investigations have been performed to prove the possible cause of decreased autofluorescence: thickened cell layers, decreased amount of flavin in tissue [9], but no definite conclusion has yet been obtained. In the present study, the diagnostic rate for cancerous lesions is almost same by conventional bronchoscopy and the LIFE system (Only one lesion was missed by conventional fiberoptic bronchoscopy). However the extent of the lesion could

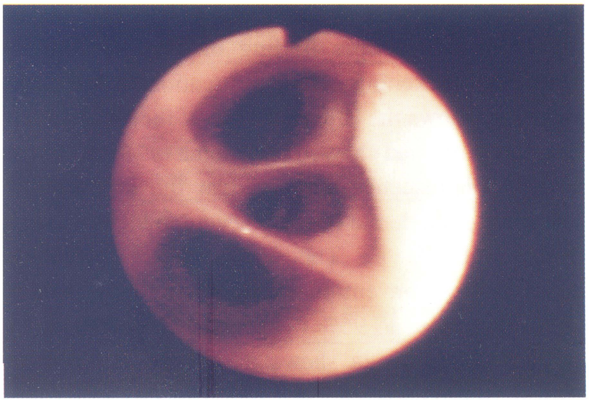

FIGURE 2A Right basal bronchus under conventional bronchoscopy. No abnormality was found.

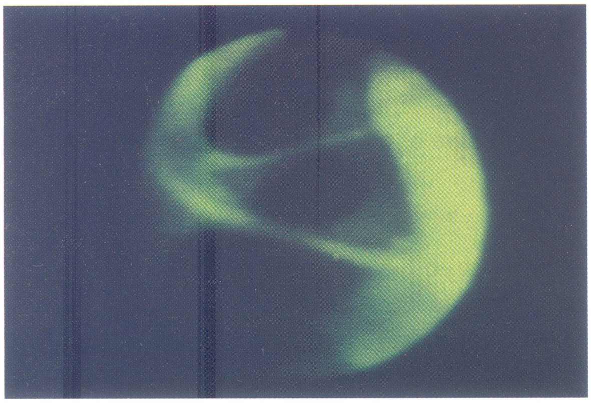

FIGURE 2B Autofluorescence from normal area was represented by green. A small cold spot was detected in the center of the bifurcation

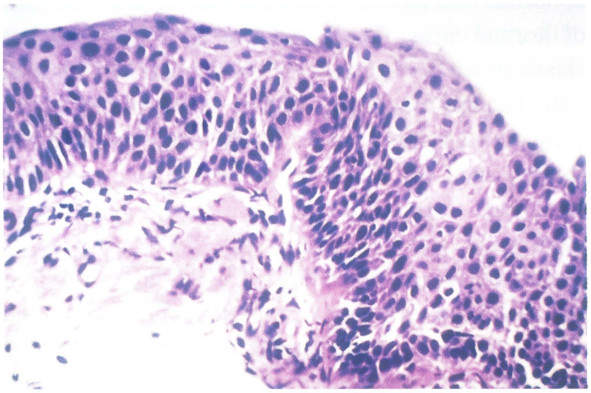

FIGURE 2C Biopsy of this area revealed to be moderately atypical squamous metaplasia.

easily be evaluated by the LIFE system, which is helpful in the preoperative determination of the resection line or the extent of photoradiation when performing photodynamic therapy. Certain areas of metaplasia develop cancer and this theory was confirmed by experiment in dogs [10]. However endoscopic findings of metaplasia have not been intensively studied. Thickening of the bifurcation with a whitish color is the only endoscopically recognizable sign of metaplasia. The sensitivity of metaplasia detection by conventional fiberoptic bronchoscope and LIFE was 28% and 96%, respectively.

Lam, a pioneer of this system, reported the sensitivity to be 40% by white light endoscopy and 91% by LIFE [7]. Concerning this point, the LIFE system is useful to estimate the effect of chemopreventive treatment. A total of 36 normal/inflamed sites existed in this study. Using the LIFE system, 16 sites were diagnosed as abnormal. Pathological examination revealed that 5 sites demonstrated truly normal histology and 11 sites demonstrated chronic inflammation. False positive results due to inflammation (basal cell hyperplasia, thickened basement membrane) in 11 sites resulted from autofluorescence being absorbed and diminished by thickened cell layers. The reason for false positive result in the remaining 5 normal sites was not clear. Lam reported that some false positive auto fluorescence lesions appeared to progress rapidly after the examination while some false negative CIS cases regressed spontaneously during follow-up [7]. It is possible biological behavior may have some influence on autofluorescence or endogenous fluoropores.

Our study suggests that fluorescence endoscopy is useful in the detection of early lung cancer as well as in the detection of precancerous lesions not detectable by conventional methods. This method requires no photosensitizer or some specific contrast. On the average, the fluorescence examination took another 10 minutes after the standard bronchoscopy procedure so that this modality could be routinely used. Fluorescence endoscopy should contribute to the detection of early stage lung cancer and therefore to the improvement of prognosis of this disease, as well as functioning as a method to investigate the pathogenesis of pulmonary disease.

### Acknowledgements

This study was supported by the Japanese Foundation for Research and Promotion of Endoscopy.

### References

[1] Woolner, L.B., Fontana, R.S., Cortese, D.A. *et al.* Roentogeographicaly occult lung cancer: Pathologic findings and frequency of multicentricity during a 10-year period. *Mayo. Clin. Proc.* 1984; **59**: 453–466.

[2] Kato, H. and Cortese, D.A. Early detection of lung cancer by means of hematoporphyrin derivative fluorescence and laser photoradiation. *Clin. Chest Med.* 1985; **6**: 237–253.

[3] Kato, H., Imaizumi, T., *et al.* Photodynamic diagnosis in respiratory tract malignancy using an eximer dye laser system. *J. Photochem. Photobiol.* 1990; **6**: 189–196.

[4] Hayata, Y., Kato, H., Konaka, C., *et al.* Photodynamic therapy in early stage lung cancer. *Lung Cancer* 1993; **9**: 287–294.

[5] Lam, S., MacAulay, C., Hung, J., *et al.* Detection of dysplasia and carcinoma *in situ* using a lung imaging fluorescence endoscope (LIFE) device. *J. Thorac. Cardiovasc. Surg.* 1993; **105**: 1035–1040.

[6] Palcic, B., Lam, S., Hung, J., *et al.* Detection and localization of early lung cancer by imaging techniques. *Chest* 1991; **99**: 742–743.

[7] Lam, S., MacAulay, C., LeRiche, J.C., *et al. Early localization of bronchogenic carcinoma. Diagnostic and Therapeutic Endoscopy.* 1994; **1**: 75–78.

[8] Hung, J., Lam, S., LeRiche, J.C., *et al.* Autofluorescence of normal and malignant bronchial tissue. *Lasers Surg. Med.* 1991; **11**: 99–105.

[9] Furuya, T., Ikeda, N., Okada, S., *et al.* Autofluorescence of bronchial tissue. *J. of Japan Society for Laser Medicine* 1996; **17**: 75–80. (in Japanese)

[10] Konaka, C., Auer, G., Nasiell, M., *et al.* Sequential cytomorphological and cytochemical changes during development of bronchial carcinoma in beagle dogs exposed to 20-methylcholanthrene. *Acta Histochem. Cytochem.* 1982; **15**: 779–797.

